# Postmarketing safety and effectiveness of recombinant factor IX (nonacog alfa) in Japanese patients with haemophilia B

**DOI:** 10.1111/hae.13783

**Published:** 2019-06-06

**Authors:** Katsuyuki Fukutake, Masashi Taki, Tadashi Matsushita, Michio Sakai, Ami Takata, Hiromi Yamaguchi, Toshiyuki Karumori

**Affiliations:** ^1^ Department of Laboratory Medicine Tokyo Medical University Tokyo Japan; ^2^ Department of Molecular Genetics of Coagulation Disorders Tokyo Medical University Tokyo Japan; ^3^ Department of Pediatrics, St. Marianna University School of Medicine Yokohama City Seibu Hospital Yokohama Japan; ^4^ Department of Transfusion Medicine Nagoya University Hospital Nagoya Japan; ^5^ Department of Pediatrics University of Occupational and Environmental Health Kitakyushu Japan; ^6^ Pfizer Japan Tokyo Japan

**Keywords:** effectiveness, haemophilia B, Japan, nonacog alfa, real‐world, safety

## Abstract

**Introduction:**

In 2010, nonacog alfa became the first recombinant factor IX (rFIX) available in Japan for patients with haemophilia B.

**Aim:**

To determine real‐world safety (adverse events, incidence of inhibitors) and effectiveness of nonacog alfa in Japan.

**Methods:**

This multicentre, prospective, observational, postmarketing surveillance study enrolled previously treated and untreated patients (PTPs and PUPs, respectively) who were observed for 1 and 2 years, respectively, after initiating nonacog alfa therapy. Safety and effectiveness were assessed for each treatment type. Annualized bleeding rate (ABR) and incremental recovery of rFIX were also evaluated.

**Results:**

Overall, 312 of 314 patients enrolled from 173 sites were eligible for the safety analysis set (PTPs, 281; PUPs, 28; other, 3). Mean age was 25.4 (PTPs) and 14.8 (PUPs) years. Haemophilic severity ranged from mild to severe, and 133 (42.6%) patients had haemophilic arthropathy. Of 285 patients (PTPs, 257; PUPs, 28) in the effectiveness set, 112 received on‐demand treatment for 1161 bleeding episodes (effectiveness rate, 93.7%) and 185 received routine prophylaxis (effectiveness rate, 95.5%). No spontaneous bleeding was observed in 52.4% of patients during prophylactic treatment. Median ABR was lower during routine prophylaxis (2.0) vs the rest of the observation period (8.3). A weak negative correlation was found between body weight and the reciprocal of rFIX recovery. Eleven adverse drug reactions occurred in 7 PTPs (2.2% [7/312]); recurrence of inhibitor was observed in 1 patient, but no new inhibitor developed in PTPs or PUPs.

**Conclusion:**

Nonacog alfa therapy is safe and effective in the real‐world scenario in Japan.

## 1 INTRODUCTION

Haemophilia B is a hereditary coagulation disorder caused by deficiency or dysfunction of blood‐clotting factor IX (FIX). In Japan, 1097 patients were reported to have haemophilia B in 2016.^1^ Bleeding episodes in patients with haemophilia B are treated by FIX replacement. Routine prophylaxis is also widely adopted and involves regular FIX replacement therapy, aiming to maintain FIX levels to prevent bleeding.^2^, ^3^, ^4^, ^5^, ^6^

Nonacog alfa (BENEFIX^®^, Pfizer, New York, NY, USA), a coagulation FIX product, is a purified protein produced by recombinant DNA technology for use in haemophilia B therapy. It was approved in the United States and Europe in 1997 and is currently marketed in more than 50 countries worldwide. In Japan, Wyeth Pharmaceuticals Inc. (currently Pfizer Inc.) obtained market authorization of nonacog alfa for control and prevention of bleeding events in patients with haemophilia B (congenital blood coagulation FIX deficiency) in 2009. As nonacog alfa is the first recombinant FIX (rFIX) product available in the local market since 2010 and only a limited number of patients had been enrolled in domestic clinical trials, the Japanese regulatory authority (Pharmaceuticals and Medical Devices Agency) required Pfizer Japan to conduct a postmarketing surveillance (PMS) study as an approval condition.

In the last couple of years, several extended half‐life blood coagulation factor products gained regulatory approval for the treatment of haemophilia B.^7^ However, access to this most advanced treatment option remains limited to developed countries,^8^ and the need for standard half‐life recombinant or plasma‐derived FIX products is still high.

The objectives of this study were, therefore, to determine the real‐world safety, including occurrence of adverse events (AEs) and incidence of inhibitors, and effectiveness of nonacog alfa among patients with haemophilia B in Japan.

## 2 MATERIALS AND METHODS

### 2.1 Study design and patients

This multicentre, prospective, observational, PMS study was conducted in accordance with the Japanese regulatory requirements stipulated in the Good Post‐Marketing Study Practice (GPSP). The enrolment period was from January 2010 to December 2014 (5 years after product launch), during which all patients treated with nonacog alfa across Japan were to be enrolled into the study. All enrolled patients were followed up by their physicians in routine practice for 1 year after initiating nonacog alfa therapy for previously treated patients (PTPs) and 2 years for previously untreated patients (PUPs, ie patients who had previously received blood coagulation FIX products other than nonacog alfa for no longer than 3 exposure days). The target number of patients was 300, including 20 PUPs. Safety, effectiveness and FIX recovery data were collected every 6 months for each patient using case report forms completed by the physician investigators. There were no specified activities, visits or investigations in the study.

### 2.2 Safety assessment

The primary analysis was summarized using the number and incidence rate of adverse drug reactions (ADRs) according to the severity, action taken, seriousness, outcome and causality of AE to nonacog alfa. The safety analysis set (SAS) comprised patients who satisfied the eligibility criteria and received at least one dose of nonacog alfa. AEs and ADRs were tabulated according to system organ class and preferred terms of the Medical Dictionary for Regulatory Activities/Japanese (MedDRA/J) version 17.0.

### 2.3 Effectiveness assessment

The effectiveness analysis set (EAS) comprised patients from the SAS with effectiveness evaluated at least once after infusion with nonacog alfa, excluding those with protocol violation, no visit after the first day of treatment, other than target disease and non‐assessable effectiveness. Effectiveness of nonacog alfa infusion used for routine prophylaxis, on‐demand treatment and surgical prophylaxis was rated by the physicians vs their expectations using a 4‐point scale of ‘excellent’, ‘good’, ‘moderate’ or ‘no response’ (Appendix S1). The annualized bleeding rates (ABRs) were also analysed to objectively assess the effectiveness of nonacog alfa.

#### 2.3.1 rFIX recovery

The incremental recovery of rFIX ([IU/dL]/[IU/kg]) was calculated as the observed postinfusion increase of FIX activity (IU/mL) in plasma divided by the rFIX dosage. The recommended conditions for assessment of recovery were evaluation of FIX levels in the non‐haemorrhagic period at first‐time rFIX replacement, a washout period of ≥4 days and 2 measurements that were recorded before and 30 minutes after infusion.

### 2.4 Statistics

#### 2.4.1 Correlation coefficient of FIX level

The correlation of body weight and age with reciprocal of observed incremental FIX recovery was assessed using a scatter plot with a non‐parametric regression based on locally weighted scatter plot smoothing. In each plot, Spearman's rank correlation coefficient was calculated and the test for non‐correlation was performed.

#### 2.4.2 Effectiveness

Clinical effectiveness rate, with the corresponding exact 2‐sided 95% confidence interval, was defined as the percentage of evaluations assessed as clinically effective (excellent or good) over the total number of evaluations in each treatment type. Patient‐based calculation of effectiveness evaluation was also performed. The most frequent evaluation result or the lower result (in case of same frequency) of effectiveness (4‐point scale) in each patient was defined as ‘the mode of effectiveness’ and used as a representative value for each patient. The final assessment of routine prophylaxis for each patient was also used as a representative value of the effectiveness of routine prophylaxis.

Patients were stratified by haemophilic severity, and the ABR was calculated for those with and without haemophilic arthropathy after dividing their observation period into routine prophylaxis period and rest of the observation period. Any divided treatment period lasting for <7 days was excluded from the ABR calculation.

## 3 RESULTS

### 3.1 Patient flow and baseline characteristics

**Figure 1:**
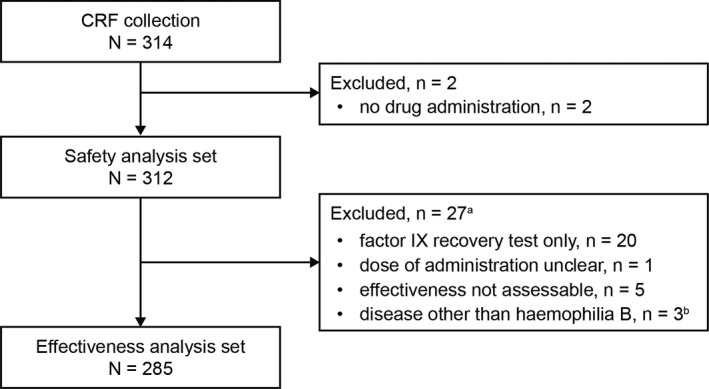
Patient flow chart. ^a^Reasons for exclusion are counted in duplicate. ^b^Bleeding, haemophilia B carrier and liver cirrhosis (n = 1 each). CRF, case report form

**Figure 2:**
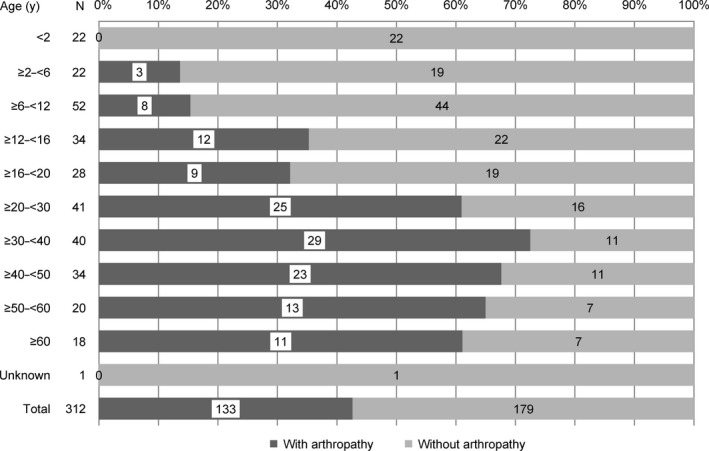
Haemophilic arthropathy at baseline summarized by age category

Overall, 314 patients were enrolled at 173 sites from January 2010 to January 2012; of these, two were excluded from the SAS because of no drug administration. A majority of patients in the SAS were PTPs (281/312 [90.1%]). Overall, 285 patients (PTPs, 257 [90.2%]; PUPs, 28 [9.8%]) were included in the EAS (Figure 1, Table 1). In the SAS (mean age: 24.5 years [PTPs, 25.4 years; PUPs, 14.8 years]), deficiency was severe (<1% FIX activity level) in approximately half of the PTPs (142/281 [50.5%]) and PUPs (14/28 [50.0%]) (Table 1). At baseline, 133 (42.6%) patients had haemophilic arthropathy; the most common joints involved were ankles, followed by knees and elbows. More than 60% of patients had arthropathy in the age groups of ≥20 years (Figure 2).

**Table 1:** Patient baseline characteristics

Patient background	Safety analysis set (N = 312)	Effectiveness analysis set (N = 285)
Age, y		
n	311	284
Mean (SD)	24.5 (18.6)	23.9 (18.3)
Median (minimum, maximum)	19.0 (1 mo, 74 y)	18.5 (1 mo, 74 y)
Age category, n (%)		
<15 y	124 (39.7)	116 (40.7)
15 to <65 y	179 (57.4)	163 (57.2)
≥65 y	8 (2.6)	5 (1.8)
Unknown	1 (0.3)	1 (0.4)
Sex, n (%)		
Male	308 (98.7)	282 (98.9)
Female	4 (1.3)	3 (1.1)
Body weight, kg		
n	300	274
Mean (SD)	48.2 (21.1)	47.4 (21.2)
Median (minimum, maximum)	53.8 (2.4, 106.0)	52.9 (2.4, 106.0)
Severity (% FIX activity level), n (%)		
Mild (5% to <40%)	43 (13.8)	40 (14.0)
Moderate (1% to <5%)	98 (31.4)	91 (31.9)
Severe (<1%)	156 (50.0)	142 (49.8)
Unknown	15 (4.8)	12 (4.2)
Previous treatment history, n (%)		
PTPs [Severity: Mild, Moderate, Severe, Unknown]	281 (90.1) [31, 95, 142, 13]	257 (90.2) [29, 88, 128, 12]
PUPs [Severity: Mild, Moderate, Severe]	28 (9.0) [11, 3, 14]	28 (9.8) [11, 3, 14]
Other	3 (1.0)	0
History of allergy, n (%)		
Yes	42 (13.5)	38 (13.3)
No	266 (85.3)	244 (85.6)
Unknown	4 (1.3)	3 (1.1)
History of FIX inhibitors before nonacog alfa administration, n (%)		
Yes	6 (1.9)	4 (1.4)
No	279 (89.4)	257 (90.2)
Not determined	27 (8.7)	24 (8.4)
Haemophilic arthropathy, n (%)^a^		
Yes	133 (42.6)	119 (41.8)
No	179 (57.4)	166 (58.2)
Comorbidities, n (%)		
Abnormal liver function	96 (30.8)	87 (30.5)
Hepatitis B	10 (3.2)	10 (3.5)
Hepatitis C	93 (29.8)	85 (29.8)
Other	8 (2.6)	6 (2.1)
HIV infection	49 (15.7)	44 (15.4)

Note

The percentage values are rounded off to 1 decimal place and may not add up to 100%.

Abbreviations: FIX, factor IX; HIV, human immunodeficiency virus; PTP, previously treated patient; PUP, previously untreated patient; SD, standard deviation.

a Diagnosis of haemophilic arthropathy was based on investigators’ discretion.

### 3.2 Nonacog alfa administration

Overall, there were 18 292 nonacog alfa infusions during the study period (PTPs, 16 201 infusions; PUPs, 2089 infusions; patients with disease other than haemophilia B, 2 infusions). The mean ± standard deviation (SD) (25th percentile, median, 75th percentile) number of infusions was 63.3 ± 54.8 (16.5, 57.5, 100.0) in PTPs and 104.5 ± 85.4 (17.5, 133.5, 175.5) in PUPs. The purpose of administration of nonacog alfa was routine prophylaxis, on‐demand treatment, surgical prophylaxis, prophylaxis at chance (eg in advance of occasional physical exercise), short‐term prophylaxis, rFIX recovery test and immune tolerance induction (ITI).

Patients in the SAS (N = 312) were categorized into five groups by treatment method (Table 2); of these, nearly half (47.4%) received nonacog alfa as routine prophylaxis, a quarter (24.0%) as on‐demand treatment and one‐eighth (12.2%) as treatment switched.

**Table 2:** Patient groups according to treatment method

Patient group		Method of treatment	Safety analysis set (N = 312) n (%)	Effectiveness analysis set (N = 285) n (%)
1	Routine prophylaxis	Routine prophylaxis only	136 (43.6)	135 (47.4)
		Routine prophylaxis and other replacement^a^	12 (3.8)	12 (4.2)
		Total	148 (47.4)	147 (51.6)
2	Treatment switched	Routine prophylaxis and on‐demand treatment with/without other replacement^a^	38 (12.2)	38 (13.3)
		Total	38 (12.2)	38 (13.3)
3	On‐demand treatment	On‐demand treatment only	46 (14.7)	45 (15.8)
		On‐demand treatment and other replacement^a^	29 (9.3)	29 (10.2)
		Total	75 (24.0)	74 (26.0)
4	Other treatment	Surgical prophylaxis only	14 (4.5)	12 (4.2)
		Surgical prophylaxis and Prophylaxis at chance	2 (0.6)	2 (0.7)
		Prophylaxis at chance only	14 (4.5)	12 (4.2)
		Immune tolerance induction only	1 (0.3)	0
		Total	31 (9.9)	26 (9.1)
5	Recovery test	FIX recovery test only	20 (6.4)	0

Abbreviation: FIX, factor IX.

a Other replacement: surgical prophylaxis, prophylaxis at chance and short‐term prophylaxis. Patients in every group may be tested rFIX (recombinant factor IX) recovery. The percentage values are rounded off to 1 decimal place and may not add up to 100%.

**Figure 3:**
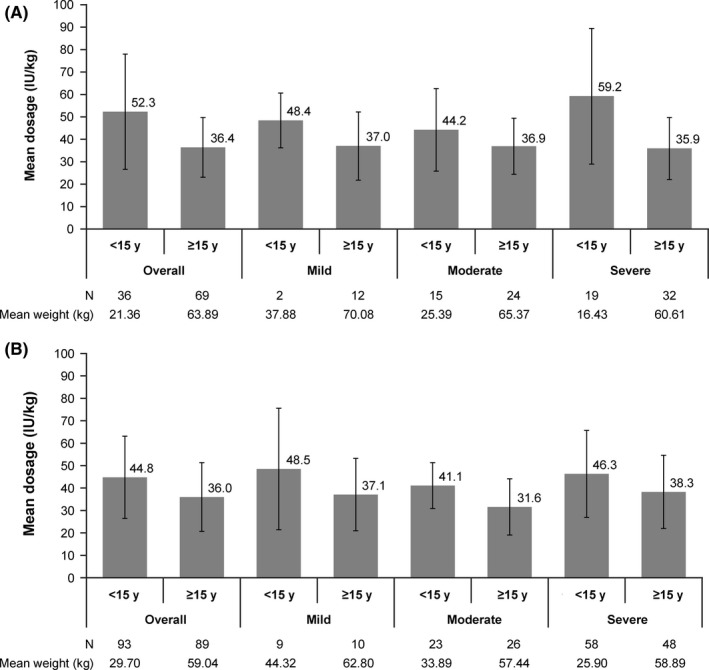
Nonacog alfa dose (IU/kg; mean ± SD) in on‐demand treatment (A) and routine prophylaxis (B). A, Of 113 patients who underwent on‐demand treatment, eight were excluded from calculation of mean single dosage because there was no record of dosage (n = 1), body weight (n = 6) or age (n = 1). B, Of 186 patients who underwent routine prophylaxis, four patients were excluded from calculation of mean single dosage because there was no record of body weight. Eight patients with unknown severity were included in the overall group but excluded from mean dosage stratified by severity. SD, standard deviation

The mean single dosage of nonacog alfa for on‐demand treatment in 106 patients was 41.8 IU/kg. To assess the difference in the mean single dosage of nonacog alfa between age groups, patients were divided into two groups (<15 years and ≥15 years) as per general practice in Japanese pharmaceutical affairs. Patients aged <15 years received numerically higher dosages vs those aged ≥15 years (52.3 vs 36.4 IU/kg), both overall and when stratified by severity (Figure 3A). The mean single dosage of nonacog alfa in 182 patients receiving routine prophylaxis was 40.5 IU/kg (44.8 and 36.0 IU/kg in patients aged <15 and ≥15 years, respectively). When summarized by severity, the mean dosage in patients aged <15 years vs those aged ≥15 years was 48.5 vs 37.1 IU/kg (mild), 41.1 vs 31.6 IU/kg (moderate) and 46.3 vs 38.3 IU/kg (severe), respectively (Figure 3B).

Of the 186 patients who received routine prophylaxis, 100 patients (53.8%) received 2.0 to <3.0 infusions per week and 57 (30.6%) received 1.0 to <2.0 infusions per week. The mean ± SD (25th percentile, median, 75th percentile) number of infusions, excluding three patients with unknown infusion frequency, was 1.8 ± 0.8 (1.0, 2.0, 2.0) per week.

### 3.3 Safety

In the SAS (312 patients), 11 ADRs were observed in 7 PTPs (2.24%). One serious ADR of recurrence of anti‐FIX antibody positive was observed in a PTP (0.32%), and 1 unexpected ADR of epilepsy was observed in a PTP (0.32%). Other non‐serious ADRs were headache, dizziness, cough, dyspnoea, nausea, rash, urticaria and injection site pruritus. No thrombotic events were observed. No ADRs including new inhibitor development were observed among the 28 PUPs despite their high mean number (>100) of infusions (Table 3). The patient who showed recurrence of anti‐FIX antibody positive already had FIX inhibitor at enrolment. Moreover, his inhibitor recurred during ITI with nonacog alfa after 4 months of inhibitor absence. In this patient, the inhibitor titre was >5 BU/mL when he was treated with plasma‐derived FIX before enrolment. At baseline, the titre was 1 BU/mL, and this patient had history of allergy such as skin pruritus and urticaria but did not have history of anaphylaxis or nephrosis. During the course of the ITI with nonacog alfa, after the recurrence of anti‐FIX antibody positive, the inhibitor titre was elevated to 11 BU/mL at the maximum.

**Table 3:** Adverse drug reactions

Events	PTPs (n = 281)	PUPs (n = 28)	Other diseases (n = 3)	Overall (N = 312)
Number of patients with ADRs	7 (2.49)	0 (0.00)	0 (0.00)	7 (2.24)
Number of events, n	11	0	0	11
Dizziness	1 (0.36)	0 (0.00)	0 (0.00)	1 (0.32)
Epilepsy	1 (0.36)	0 (0.00)	0 (0.00)	1 (0.32)
Headache	2 (0.71)	0 (0.00)	0 (0.00)	2 (0.64)
Cough	1 (0.36)	0 (0.00)	0 (0.00)	1 (0.32)
Dyspnoea	1 (0.36)	0 (0.00)	0 (0.00)	1 (0.32)
Nausea	1 (0.36)	0 (0.00)	0 (0.00)	1 (0.32)
Rash	1 (0.36)	0 (0.00)	0 (0.00)	1 (0.32)
Urticaria	1 (0.36)	0 (0.00)	0 (0.00)	1 (0.32)
Injection site pruritus	1 (0.36)	0 (0.00)	0 (0.00)	1 (0.32)
Anti‐FIX antibody positive	1 (0.36)	0 (0.00)	0 (0.00)	1 (0.32)

Note

Data are shown as n (%) unless otherwise stated. Summarized by preferred term according to Medical Dictionary for Regulatory Activities/Japanese version 17.0.

Abbreviations: ADR, adverse drug reaction; FIX, factor IX; PTP, previously treated patient; PUP, previously untreated patient.

### 3.4 Effectiveness

#### 3.4.1 On‐demand treatment

In the EAS, 112 patients (PTPs, 97; PUPs, 15) received on‐demand treatment with nonacog alfa for 1161 bleeding episodes (PTPs, 1063; PUPs, 98), of which 925 were spontaneous (PTPs, 895; PUPs, 30), 230 were traumatic (PTPs, 162; PUPs, 68), and 6 were unknown (PTPs, 6; PUPs, 0). On average, 1.8 infusions were required to control these bleeding episodes.

For the 1161 bleeding events observed during the study period, 671 effectiveness assessments were performed by the physicians. The effectiveness based on each assessment was evaluated as excellent in 224 cases (33.4%), good in 405 (60.4%), moderate in 36 (5.4%), no response in 2 (0.3%) and not evaluable in 4 (0.6%). The overall clinical effectiveness rate was 93.7%.

**Figure 4:**
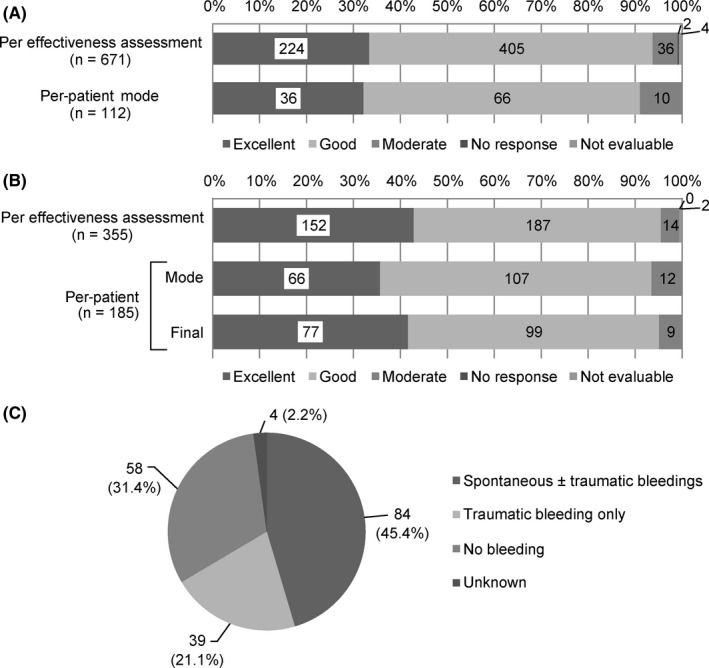
Effectiveness outcome of on‐demand treatment (A) and routine prophylaxis (B) and bleeding episodes in patients receiving routine prophylaxis (C). The percentage values are rounded off to 1 decimal place and may not add up to 100%

The effectiveness based on the mode of effectiveness per patient, corresponding to 1 assessment per patient, was evaluated as excellent in 36 patients (32.1%), good in 66 (58.9%), moderate in 10 (8.9%) and no response in 0 patients (0.0%). The overall clinical effectiveness based on per‐patient mode was 91.1% (Figure 4A).

#### 3.4.2 Routine prophylaxis

In the EAS, 185 patients (PTPs, 172; PUPs, 13) received nonacog alfa as routine prophylaxis. The severity of the disease at baseline was mild in 19 patients (10.3%), moderate in 51 patients (27.6%), severe in 105 patients (56.8%) and unknown in 10 patients (5.4%). Among these 185 patients, 355 effectiveness assessments were performed by the physicians. The effectiveness based on each assessment was evaluated as excellent in 152 instances (42.8%), good in 187 (52.7%) and moderate in 14 (3.9%). The overall clinical effectiveness rate was 95.5%.

The effectiveness based on the mode of effectiveness per patient was evaluated as excellent in 66 patients (35.7%), good in 107 (57.8%), moderate in 12 (6.5%) and no response in 0 patients (0.0%). The overall clinical effectiveness based on per‐patient mode was 93.5% and that in PUPs was 100%.

Based upon the physician’s effectiveness analysis from the final assessment of each patient, outcomes were deemed excellent in 77 patients (41.6%), good in 99 (53.5%), moderate in 9 (4.9%) and no response in 0 patients (0.0%). Overall clinical effectiveness rate was 95.1% (Figure 4B).

Almost one‐third of the patients (31.4%, 58/185) among those who received routine prophylaxis had no bleeding events during the prophylactic treatment period (Figure 4C). Although bleeding occurred in 123 (66.5%) patients, 39 (21.1%) had traumatic bleeding only and 84 (45.4%) had any bleeding events, including spontaneous bleeding. Almost half (52.4%, 97/185) of patients who received prophylaxis had no spontaneous bleeding during their prophylaxis treatment period.

#### 3.4.3 Surgical prophylaxis (including dental procedures)

In the EAS, the effectiveness of surgical prophylaxis (including dental procedures) was evaluated in 28 surgeries in 21 patients (PTPs, 12; PUPs, 9). Effectiveness outcomes, with respect to blood loss, were deemed excellent in 10 events (35.7%) and good in 18 (64.3%). The overall clinical effectiveness rate was 100.0%. Six events (tooth extraction, hepatic resection, pulmonary artery plasty, laparoscopic cholecystectomy, artificial joint replacement in the left knee and artificial femoral head implantation) required blood transfusions.

#### 3.4.4 Annualized bleeding rate

**Figure 5:**
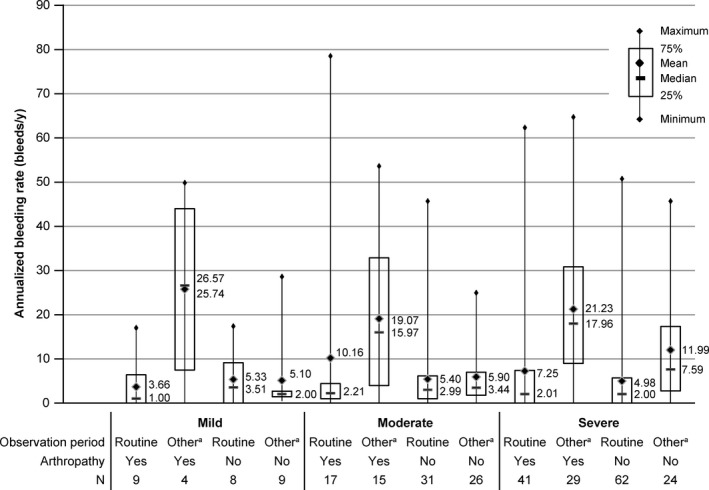
Annualized bleeding rate summarized by existence of haemophilic arthropathy and severity of haemophilia (effectiveness analysis set). Any treatment period (routine or other) lasting for <7 days was excluded from the calculation of annualized bleeding rate. ^a^Surgical prophylaxis, prophylaxis at chance, short‐term prophylaxis and FIX recovery test. FIX, factor IX

The ABR was numerically lower in the routine prophylaxis period (mean ± SD: 6.0 ± 11.3; 25th percentile, median, 75th percentile: 0.0, 2.0, 6.0) compared with the rest of the observation period (mean ± SD: 13.7 ± 15.0; 25th percentile, median, 75th percentile: 2.0, 8.3, 21.0). This difference was prominent among patients with severe haemophilia B or haemophilic arthropathy (Figure 5).

#### 3.4.5 rFIX recovery

**Figure 6:**
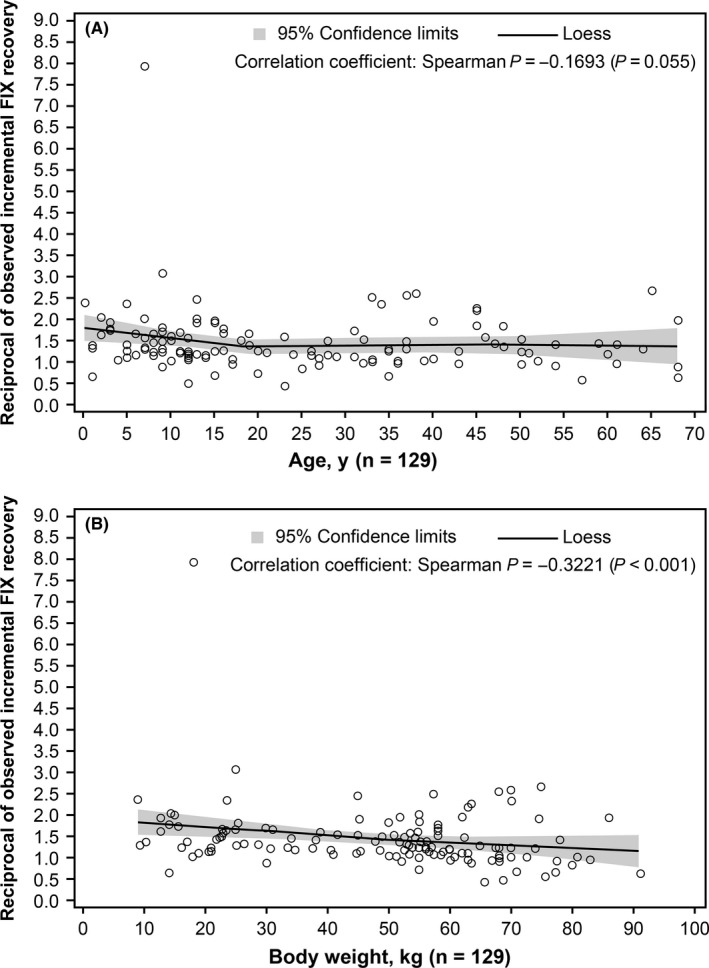
Correlation between reciprocal of observed incremental FIX recovery and age (A)/body weight (B). FIX, factor IX; Loess, locally weighted scatter plot smooth

The increased value of FIX was measured under recommended conditions in 129 events; corresponding median and mean (SD) reciprocal of recovery values were 1.284 and 1.449 (0.748), respectively. Correlation between reciprocal of observed incremental FIX recovery and age was not significant (*P *= 0.055; Figure 6A). However, a weak, significant (negative) correlation was observed between the reciprocal of observed incremental FIX recovery and body weight; increase in body weight led to decrease in recovery (*P *< 0.001; Figure 6B).

## 4 DISCUSSION

Although the present study was conducted in accordance with the Japanese regulatory requirements, useful information on the treatment of haemophilia B in Japan was collected. The enrolment of 314 patients over a 2‐year period indicates that nonacog alfa use spread promptly after approval as it was the first rFIX product available in Japan. According to the Japanese national survey of haemophilia, 819 patients were reported to have haemophilia B as of May 2012, and 177 of 481 patients (36.8%) had been treated with routine prophylaxis.^9^ In this study, almost 60% (186/312) of the patients were treated with routine prophylaxis and the rate was considerably higher than that of usual haemophilia B populations at the time of research. It is thus conceivable that the patient population who have preferred nonacog alfa have had comparatively advanced knowledge of treatments concerning safety of recombinant products (ie minimizing the risk of blood‐borne pathogen transmission) and efficacy of prophylaxis (ie preventing bleeding events).^10^

Dosage per body weight in patients aged ≥15 years was numerically lower than that in patients aged <15 years, as illustrated in Figure 3. Although this may be attributable to the high clearance and low recovery of FIX in young patients, it is more likely due to the influence of the available formulations during the study period (500, 1000 and 2000 IU/vial). The reason for the lower doses in patients aged ≥15 years is unclear, but some patients or physicians might have preferred one vial dosing for the convenience of injection even if the optimal dosage for the patients would be higher than 2000 IU. Currently, 3000 IU vials are available and it is expected that adequate dosage will now be achieved in patients who need high dosages.

Results demonstrated that nonacog alfa is a well‐tolerated option for managing patients with haemophilia B. No new inhibitor antibodies were identified in this study population (patients with severe deficiency; 142/281 PTPs, 14/28 PUPs), and our results are consistent with those of previous studies where the incidence of inhibitor antibody development in haemophilia B has been reported as 1%–5%.^11^, ^12^ However, although PUPs had more than 100 infusions on average in this study, the results should be interpreted with caution as administration to a limited number of PUPs is not sufficient to draw conclusions about whether nonacog alfa carries less risk of inhibitor development vs other agents.

In treatment with nonacog alfa for routine prophylaxis, on‐demand treatment and surgical prophylaxis, physicians’ evaluations of effectiveness were consistently favourable for each purpose. Further, the low ABR during routine prophylaxis compared with the rest of the observation period indicates the effect of prophylaxis treatment to prevent bleeding. In particular, the noticeable difference in ABR in patients with severe haemophilia or haemophilic arthropathy may confirm the benefit of routine prophylaxis for such patients.

However, the median ABR was 2.0 even during the routine prophylaxis treatment period, and bleeding occurred in 66.5% of patients receiving prophylaxis. Although various factors such as incomplete adherence to the prophylaxis regimen may be involved, this could indicate that the clinical practice prophylaxis protocol may have been insufficient to completely prevent bleeding. This may be due to the fact that a standardized prophylaxis regimen recommended by guidelines based on the traditional theory to maintain plasma FIX level above 1% was implemented.^13^ The administration regimen of routine prophylaxis should be tailored to each patient's bleeding symptoms as well as their lifestyle, such as intensity, frequency and timing of their activities. This flexible personalized treatment may further improve the effectiveness of routine prophylaxis treatment. Furthermore, although a statistically significant negative relationship between reciprocal of FIX recovery and body weight was observed, there was a large difference in recovery rate between individuals. This also supports the importance of measuring FIX recovery for each patient to determine the necessary dosage for on‐demand treatment of bleeding as well as the appropriate prophylaxis regimen for each patient.

Our results demonstrated that nonacog alfa has a favourable risk‐benefit profile in the control and prevention of bleeding events in patients with haemophilia B in Japan. As such, we suppose that nonacog alfa is still an important treatment option especially to maintain surgical operations and for patients with intense activity or those who need routine prophylaxis when access to extended half‐life products is limited. Nonacog alfa can also be a significant alternative to plasma‐derived FIX products, as it is a recombinant protein product and thus can minimize the risk of transmission of blood‐borne pathogens.

### 4.1 Limitations

Although results of this study should reflect real‐world use of nonacog alfa, interpretation of this study is limited as it was implemented as a GPSP‐compliant, all‐patient, non‐interventional, observational study. Also, the ABR between the routine prophylaxis period and rest of the observation period was compared in a non‐randomized manner, and the study was not designed to assess the differences in ABR.

## 5 CONCLUSION

Results of this PMS study show that nonacog alfa therapy is safe and effective in the real‐world scenario in Japan. The results suggest that nonacog alfa was well tolerated and appropriately used under routine clinical practice as on‐demand, routine prophylaxis and in perioperative management of haemophilia B patients.

## CONFLICT OF INTEREST

KF reports grants and personal fees from Baxter/Baxalta, Bayer, Pfizer, CSL Behring, Novo Nordisk, Biogen/Bioverativ and Kaketsuken; grants from Japan Blood Products Organization and Ortho Clinical Diagnostics; personal fees from SRL, LSI Medience, Roche Diagnostics, Siemens, Sekisui Medical, Fujirebio, Abbott, Torii Pharmaceuticals, Octapharma, Chugai Pharmaceutical and BioMarin; and fee for PMS from CMIC, outside the submitted work. MT is an advisory board member of Bioverativ, Chugai Pharmaceutical, CSL Behring and Novo Nordisk; clinical trial investigator for Bioverativ, Chugai Pharmaceutical, CSL Behring, Novo Nordisk and Octapharma; and reports personal fees from Baxalta/Shire, Bayer, Bioverativ, CSL Behring, Novo Nordisk, Pfizer, Chugai Pharmaceutical and Kaketsuken, outside the submitted work. TM reports personal fees and research funding from Novo Nordisk, Bayer, Bioverativ and CSL Behring; personal fees from Shire, Chugai Pharmaceutical and Pfizer; and research funding from JB and Kaketsuken, outside the submitted work. MS reports personal fees from Novo Nordisk, Bayer, Shire, JB, Bioverativ, CSL Behring, Chugai Pharmaceutical and Pfizer, outside the submitted work. AT and HY were full‐time employees of Pfizer Japan until November 2018 and are currently full‐time employees of Pfizer R&D Japan GK. TK is a full‐time employee of Pfizer Japan.

## AUTHOR CONTRIBUTION

KF was involved in study design, the interpretation of data and manuscript preparation. MT, TM and MS were involved in study design, the interpretation of data and revision of the manuscript. AT, HY and TK were involved in data analysis and manuscript preparation. All the authors read and approved the final version of the paper. Editorial support was provided by Dr. Annirudha Chillar of Cactus Communications, and funded by Pfizer Japan.

### DATA AVAILABILITY

The data sets analysed during the current study are not available because data sharing with third parties was not included in the contract with study sites.
