# Supplementary material for: Postmarketing safety and effectiveness of recombinant factor IX (nonacog alfa) in Japanese patients with haemophilia B
Source: Haemophilia. 2019 Jun 6;25(4):e247–56. doi: 10.1111/hae.13783 (PMC6852692; doi:10.1111/hae.13783)
Supplement: Supplementary file 1 [file HAE-25-e247-s001.docx]

**SUPPLEMENTARY MATERIAL S1** Criteria for effectiveness evaluation

The effectiveness assessment was conducted for on-demand and other treatment and routine prophylaxis using a 4-point scale of ‘excellent’, ‘good’, ‘moderate’, and ‘no response’, or ‘not evaluable’. The rating was given by the physician based on the following observation items:

1) Number of bleeding episodes during routine prophylaxis.

2) Number of doses required for bleeding to stop during a bleeding episode.

3) Subjective assessment on the effect of each administration of nonacog alfa in bleeding episodes.

**Criteria for effectiveness assessment**

**Excellent:** Completely satisfactory response (achieving a response equivalent to the best effect achievable with other blood coagulation factor IX products and comparably prompt improvement in similar bleeding or procedure).

**Good:** Satisfactory response (achieving a response equivalent to the effects frequently seen with other blood coagulation factor IX products and comparably prompt improvement in similar bleeding or procedure).

**Moderate:** Not satisfactory response (response inferior to the effects frequently seen with other blood coagulation factor IX products in similar bleeding or procedure).

**No response:** No improvement at all (haemostasis management was difficult on a daily basis without other factors causing bleeding).

The effectiveness assessment for surgical or dental procedures was conducted using the above-mentioned criteria, as well as the assessment for on-demand and other treatment and routine prophylaxis. Blood loss volume and need for blood transfusion were taken into consideration.
